# Bidirectional associations of physical activity, sleep, and self-reported mental health in young adults participating in an online wellness intervention during the COVID-19 pandemic

**DOI:** 10.3389/fpubh.2023.1168702

**Published:** 2023-05-31

**Authors:** Ryan D. Burns, Anna Bilic, Yang Bai, Timothy A. Brusseau, Julie E. Lucero, Jessica L. King Jensen

**Affiliations:** Department of Health and Kinesiology, College of Health, University of Utah, Salt Lake City, UT, United States

**Keywords:** behavior, exercise, health, mental health, sleep, young adult

## Abstract

**Purpose:**

The purpose of this study was to examine the bidirectional associations of physical activity (PA), sleep, and mental health in young adults participating in an online wellness intervention from October 2021 to April 2022.

**Methods:**

Participants were a sample of undergraduate students from one US university (*N* = 89; 28.0% freshman; 73.0% female). The intervention was a 1-h health coaching session that was delivered either once or twice by peer health coaches on Zoom during COVID-19. The number of coaching sessions was determined by random allocation of participants to experimental groups. Lifestyle and mental health assessments were collected at two separate assessment timepoints after each session. PA was assessed using the International Physical Activity Questionnaire–Short Form. Weekday and weekend sleep were assessed by two one-item questionnaires and mental health was calculated from five items. Cross-lagged panel models (CLPMs) examined the crude bidirectional associations of PA, sleep, and mental health across four-time waves (i.e., T1 through T4). To control for individual unit effects and time-invariant covariates, linear dynamic panel-data estimation using maximum likelihood and structural equation modeling (ML-SEM) was also employed.

**Results:**

ML-SEMs showed that mental health predicted future weekday sleep (*β* = 0.46, *p* < 0.001) and weekend sleep predicted future mental health (*β* = 0.11, *p* = 0.028). Although CLPMs showed significant associations between T2 PA and T3 mental health (*β* = 0.27, *p* = 0.002), no associations were observed when unit effects and time-invariant covariates were accounted for.

**Conclusion:**

Self-reported mental health was a positive predictor of weekday sleep and weekend sleep positively predicted mental health during the online wellness intervention.

## 1. Introduction

Physical activity (PA) and sleep are two health behaviors that have been shown to correlate with mental health (1–3). Mechanisms for these positive associations between health behaviors and mental health included improved stress control, lowered cortisol, healthier sleep habits, and lowered susceptibility to anxiety and depression via release of endogenous opioids and neurohormones within the central nervous system (4–7). A population that is consistently under stress during much of the year and may suffer from poor mental health are young adults enrolled in college courses (“university students”) (8, 9). This population is burdened by the academic stressors of being a university student in addition to the lifespan transition period of emerging adulthood, which often consists of significant and sudden changes in the physical and social environment (10–13). These changes can negatively affect a young adult’s emotional health and their well-being. Problems with higher stress and poorer health behaviors may have been exacerbated during the COVID-19 pandemic (9). A potential way to mitigate declines in mental health in university students is tailored health behavior programming (14–18).

Some health behavior interventions have shown evidence to improve mental health and wellbeing in university students (14, 15). Indeed, interventions that have targeted multiple behaviors have shown the strongest evidence to improve mental health (16, 17). However, past interventions that involved targeting multiple health behaviors may have yielded high levels of participant burden because of high durations or frequencies of health education or coaching sessions, burdens that for university students may have been magnified because of their demanding academic and work schedules. Having an in-person modality for intervention delivery may have also added additional burden due to increased transportation needs. We previously implemented a brief one-hour Zoom-based intervention that yielded improvements in university students’ sleep, physical activity, nutrition, stress management, and substance use during COVID-19 (14). However, other health behavior interventions delivered during COVID-19 showed null results for improving aspects of mental health in young adults. An 8-week mind–body physical activity intervention that used yoga poses and walking meditation and was delivered asynchronously online on Canvas for 10 min, 3 days per week did not significantly associate with better stress or wellbeing in a sample of college students (19). Additionally, an intervention study conducted by Philippot et al. (20) that used high-intensity interval training delivered *via* the use of online videos to a sample of university students for 4-weeks yielded no significant improvements in anxiety symptoms. Given these conflicting findings, further investigation is warranted.

Interventions that target multiple behaviors have outcomes that may intercorrelate and predict one another across time. Thus, change in one targeted behavior during an intervention could influence other targeted behaviors at a future timepoint, independent of an intervention’s influence (i.e., independent of experimental group allocation). PA and sleep are two health behaviors that can determine mental health (21, 22), however, no study has examined the longitudinal bidirectional associations among PA, sleep, and mental health during a multi-behavioral online intervention. Examining longitudinal bidirectional associations can help determine the directionality of association, help identify targets to improve health behaviors and mental health and may provide information on how multiple behavioral outcomes intercorrelate independent of an intervention’s effect. Therefore, the purpose of this study was to examine the bidirectional associations of PA, sleep, and mental health in a sample of university students who participated in an online wellness intervention delivered during the 2021–2022 academic year, which overlapped with the COVID-19 pandemic. The current study’s intervention expanded on our previous intervention implementing multiple brief (1-h) peer health coaching sessions to university students during COVID-19 using a randomized controlled research design. The details of this study are elsewhere. The present study was a secondary analysis of data obtained from the intervention that aimed to examine how the various behavioral and mental health outcomes longitudinally intercorrelated with each other across time. Given past work in this area, it was hypothesized that PA and weekday and weekend sleep would show a moderate and positive bidirectional association with self-reported mental health.

## 2. Materials and methods

### 2.1. Participants

Participants were a non-probability convenience sample of young adults recruited from one university in the western US who participated in the online peer health coaching intervention. Recruitment strategies included written and oral advertising (e.g., posters) and by peer referrals (e.g., word of mouth). Interested participants completed informed consent online followed by a screening survey used to determine study eligibility. The inclusion criterion included being a current undergraduate student enrolled at the host institution and there were no exclusion criteria for participating in this study. A total of 173 students expressed study interest and 89 students were enrolled. The analytic sample was mostly female (73.0%), not Hispanic/Latino (80.9%), was White (73.0%), had mothers who obtained at least a Bachelor’s degree (43.8%), did not participate in University athletics (89.9%), and was not in a fraternity or sorority (92.1%). More specific descriptive demographic statistics of the analyzed sample are presented in Supplementary Table S1. Participants received $10 per survey completed, for a total of $40 potentially earned during the study. All study procedures were approved by the University’s Institutional Review Board.

### 2.2. Intervention

The intervention was a one-hour program delivered by peer health coaches in a one-on-one setting on Zoom during the 2021–2022 academic year. Briefly, the program consisted of a pre-session self-administered survey which asked participants about their wellness and substance use behaviors and a one-on-one peer health coach session in which the participant received individualized feedback on their health habits and set two wellness goals (22). Online follow-up questionnaires were administered after each session.

Participants were randomly assigned to one of two possible sequences: a one coaching session sequence or a two-session sequence. Following a step-wedged randomized controlled research design, participants in the one-session sequence completed two repeated follow-up assessments prior to their coaching session and two additional follow-up assessments. Participants in the two-session sequence completed one coaching session with two follow-up assessments followed by a second coaching session with two additional follow-up assessments. Two weeks separated each follow-up assessment.

Each assessment timepoint was designated as a time wave (T) within the analyses, with T1 representing the first assessment, T2 representing the second assessment, etc. A variable indicating whether participants received one or two coaching was used as a time-invariant covariate within the statistical models. A total of 57 participants completed at least one health coach session and 21 participants completed two sessions. Group comparisons were not made given that this study was a secondary analysis of specific behavioral outcomes. Specific group comparisons will be reported within a separate study.

### 2.3. Assessments

#### 2.3.1. Physical activity

The PA variable was weekly metabolic equivalent of task minutes (MET-min) calculated from the validated International Physical Activity Questionnaire (IPAQ)-short form questionnaire (23). The MET-min was a metric of PA volume that indicated the amount of energy expended during a minute at rest. Higher MET-min scores indicated higher levels of PA. Calculated MET-min for walking, moderate PA, and vigorous PA were summed to calculate a total MET-min score that was used for data analysis.

#### 2.3.2. Sleep

Two items asked about how many hours of sleep the participants accrued during both weeknights and weekend nights. The two items were part of the same assessment questionnaire. The weekday sleep item asked “Over the last 2 weeks, what is the average amount of sleep you have gotten on a weeknight (excluding naps)? Note: Please select the response closest to your answer.” The weekend sleep item asked “Over the last 2 weeks, what is the average amount of sleep you have gotten on a weekend night (excluding naps)? Note: Please select the response closest to your answer.” Responses ranged from less than 4 h to 10 or more hours. Scores above 10 or more hours a day were dropped for analysis due to the non-linear association between sleep duration and health outcomes (24).

#### 2.3.3. Mental health

Mental Health was calculated from five items each with a 5-point response scale (1 = All the time, 5 = None of the time). The items were from the validated SF-36 Health Survey Questionnaire (25). The mental health items were the 5 items from SF-36 that were part of the wellbeing area and mental health dimension of the questionnaire, which showed a high degree of test–retest reliability (25). The items asked, “Have you been a very nervous person?,” “Have you felt so down in the dumps that nothing could cheer you up?,” “Have you felt calm and peaceful?,” Have you felt downhearted and blue?,” and “Have you been a happy person?.” Items were reversed coded where necessary so that high scores reflected good mental health. Internal consistency across the five items was acceptable with Cronbach’s alpha ranging from alpha = 0.76–0.88 across waves. The average of the five responses were used for data analysis. The 4 items on the SF-36 Health Survey Questionnaire that measured vitality were not included in the assessments.

### 2.4. Covariates

Covariates included in the adjusted analyses consisted of self-reported participant university status year (freshman, sophomore, junior, senior), sex (male, female, other/no-response), race/ethnicity, mother’s highest education level, and experimental group (one coaching session, two coaching sessions; see Section 2.2). The race/ethnicity variable was collapsed into a binary White/Non-White variable to maintain a high group sample size.

### 2.5. Statistical analysis

To examine the crude bidirectional associations of PA, sleep, and mental health, cross-lagged panel models (CLPMs) were employed with four assessment waves (Ts) across the course of the wellness intervention. CLPMs analyzed reciprocal associations between multiple observed variables measured at multiple Ts (26). The CLPMs were constructed as structural equation models using Stata’s “sem builder.” Each CLPM had three variables, PA, sleep, and mental health that were observed at the four Ts. Specific paths within each cross-lagged model included autoregressive paths of each variable associating with itself at the next T (e.g., T1 PA associating with T2 PA) and cross-lagged associations where a respective variable associated with another at the next T (e.g., T1 PA associating with T2 mental health). Standardized covariance coefficients among the three variables were computed at T1. Separate CLPMs were run using weekday and weekend sleep. Full Information Maximum Likelihood (FIML) was utilized to conduct available case analysis in the presence of missing data. Reporting of the results included the standardized regression coefficients (β-coefficients) with corresponding 95% Confidence Intervals.

CLPMs have limitations when attempting to make causal inferences because it does not effectively control for unobserved individual unit effects, which may yield errors that are correlated with predictor variables (instrumental variables problem), in addition to uncertainty about the treatment of initial conditions (27, 28). It is also difficult to control for observed time-invariant covariates using the CLPM approach. To account for these issues, linear dynamic panel-data estimation using maximum likelihood and structural equation modeling (ML-SEM) was employed (28). ML-SEM models contained a latent variable α that controlled for individual unit effects, eliminating the instrument variables problem. With ML-SEM, the initial conditions were treated as exogenous and did not need to be modeled, and coefficients for observed time-invariant predictors were estimated. For the ML-SEM models, coefficients for the effects of a predictor (x) on an outcome (y) were constrained to be equal across time. ML-SEM models were carried out in Stata using the “xtdpdml” command. Further details regarding ML-SEM and specifically the “xtdpdml” command have been communicated elsewhere (27).

Separate ML-SEM models were run using the directional associations observed from the CLPMs to determine if the associations held using the more rigorous analytical approach. Observed time-invariant covariates within the ML-SEMs included participant status year, sex, race/ethnicity, mother’s highest education level, and experimental group. FIML was still used within each ML-SEM to conduct available case analysis in the presence of missing data. Model fit was determined using the Root Mean Square Error of Approximation (RMSEA; acceptable fit ≤ 0.08, excellent fit ≤ 0.05 and *p*-close > 0.05), the Tucker-Lewis Index (TLI; acceptable fit ≥ 0.90, excellent fit ≥ 0.95), Comparative Fit Index [CFI; acceptable fit ≥ 0.90, excellent fit ≥ 0.95 and coefficient of determination (*R*^2^)] (29, 30). All analyses had an alpha level of *p* < 0.05 and were carried out using Stata version 17 statistical software package (College Station, Texas, United States).

## 3. Results

### 3.1. Descriptive statistics

Descriptive statistics of the analyzed variables at each T are communicated in Table 1. Males reported more MET-min at T2 (mean difference = 1,150 MET-min, *p* = 0.049) and females reported more weekend sleep at T2 (mean difference = 0.7 h, *p* = 0.011) and at T4 (mean difference = 0.8 h, *p* = 0.036). No other significant differences between the sexes were observed.

**Table 1:** Descriptive statistics of the analyzed variables (means and standard deviations) collected during the 2021–2022 academic school year.

Wave	Variable	Total Sample (*N* = 89) *M* (SD)	Females (*n* = 65) *M* (SD)	Males (*n* = 21) *M* (SD)
T1	Total MET-mins	2,179 (1,763)	2,006 (1,657)	2,780 (1,996)
	Weekday sleep (hours)	6.9 (1.0)	6.8 (1.10)	7.1 (1.0)
	Weekend sleep (hours)	7.7 (1.4)	7.8 (1.3)	7.3 (1.5)
	Mental health (1–5 scale)	3.6 (0.8)	3.5 (0.9)	3.6 (0.6)
T2	Total MET-mins	2,645 (2,075)	2,323 (1,970)	**3,474** ^*^ **(2,160)**
	Weekday sleep (hours)	6.9 (1.0)	7.0 (1.1)	6.8 (1.1)
	Weekend sleep (hours)	7.6 (1.1)	**7.8** ^**^ **(1.0)**	7.1 (1.1)
	Mental health (1–5 scale)	3.6 (0.9)	3.6 (0.9)	3.6 (0.9)
T3	Total MET-mins	2,669 (2,930)	2,540 (3,213)	3,012 (2,148)
	Weekday sleep (hours)	7.1 (0.9)	7.2 (0.8)	7.0 (0.9)
	Weekend sleep (hours)	7.7 (1.0)	7.8 (1.0)	7.3 (1.0)
	Mental health (1–5 scale)	3.6 (0.9)	3.5 (0.9)	4.0 (0.9)
T4	Total MET-mins	2,909 (3,567)	2,687 (3,772)	3,780 (2,793)
	Weekday sleep (hours)	7.3 (1.1)	7.4 (1.1)	7.0 (1.3)
	Weekend sleep (hours)	7.8 (1.2)	**7.9** ^*^ **(1.1)**	7.1 (1.2)
	Mental health (1–5 scale)	3.7 (1.0)	3.7 (1.1)	3.8 (0.8)

T, assessment timepoint; MET-min, weekly metabolic equivalent of task minutes; bold denotes statistical significance, ^*^p < 0.05, ^**^p < 0.01.

### 3.2. Cross-lagged panel models

**Figure 1:**
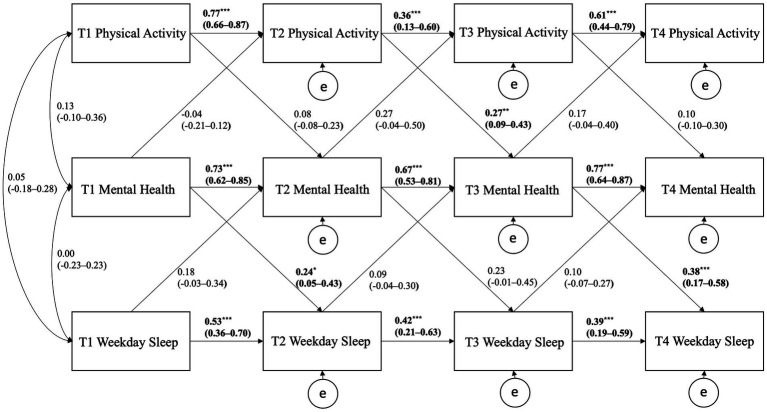
Cross-lagged panel model showing the autoregressive and cross-lagged associations of physical activity, weekday sleep, and mental health. Curved arrows are covariances; horizontal straight arrows are autoregressive direct paths; diagonal straight arrows are cross-lagged paths; e stands for error/residual in the regression equation; bold denotes statistical significance, ^*^p < 0.05, ^**^p < 0.01, ^***^p < 0.001.

**Figure 2:**
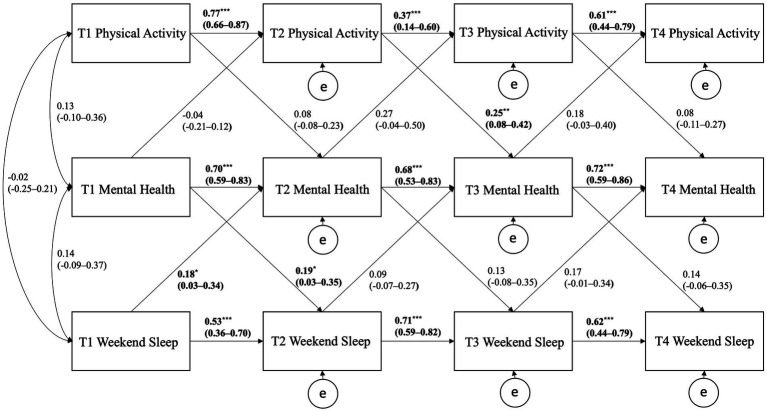
Cross-lagged panel model showing the autoregressive and cross-lagged associations of physical activity, weekend sleep, and mental health. Curved arrows are covariances; horizontal straight arrows are autoregressive direct paths; diagonal straight arrows are cross-lagged paths; e stands for error/residual in the regression equation; bold denotes statistical significance, ^*^p < 0.05, ^**^p < 0.01, ^***^p < 0.001.

Figures 1, 2 show the CLPMs for PA, weekday sleep, and mental health and for PA, weekend sleep, and mental health, respectively. For the CLPM using PA, weekday sleep, and mental health, significant cross-lagged path coefficients were observed for T2 PA positively predicting T3 mental health (*p* = 0.002), T1 mental health positively predicting T2 weekday sleep (*p* = 0.014), and T3 mental health positively predicting T4 weekday sleep (*p* < 0.001). All autoregressive paths were significant within the model but tended to be strongest for the mental health variable.

For the CLPM using PA, weekend sleep, and mental health, significant cross-lagged path coefficients were observed for T2 PA positively predicting T3 mental health (*p* = 0.004), T1 weekend sleep positively predicting T2 mental health (*p* = 0.019), and T1 mental health positively predicting T2 weekend sleep (*p* = 0.023). All autoregressive paths were significant within the model but tended to be strongest for the weekend sleep and mental health variables.

### 3.3. Dynamic panel models using maximum likelihood

The results of three ML-SEMs were reported in Table 2. Three ML-SEM models were constructed based on the results from the CLPMs with weekday sleep, weekend sleep, and mental health as respective outcomes. All models adjusted for unit effects and the time-invariant covariates of status year, sex, race/ethnicity, mother’s education level, and experimental group. An example path diagram showing the structure of the model with the weekday sleep outcome and mental health predictor was provided in Supplementary Figure S1. Using the weekday sleep outcome, mental health was a significant positive predictor of future weekday sleep (*p* < 0.001); however, using the weekend sleep outcome, mental health was not significant predictor of future weekend sleep (*p* = 0.372). Weekend sleep was the only outcome that showed significant and positive autoregressive effects across Ts (*p* < 0.001). Using the mental health outcome, weekend sleep positively predicted future mental health (*p* = 0.028); however, PA and weekday sleep did not significantly predict future mental health within the ML-SEM. ML-SEM model fit was acceptable using the weekday sleep outcome and the mental health lagged predictor (RMSEA = 0.06, 90%CI: 0.00–0.10, *p*-close = 0.345; CFI = 0.92, TLI = 0.88; *R*^2^ = 0.82), excellent for the weekend sleep outcome and the mental health lagged predictor (RMSEA = 0.00, 90%CI: 0.00–0.07, *p*-close = 0.821; CFI = 1.00, TLI = 1.00; *R*^2^ = 0.73), and acceptable using the mental health outcome with PA, weekday sleep, and weekend sleep lagged predictor variables (RMSEA = 0.06, 90%CI: 0.00–0.10, *p*-close = 0.329; CFI = 0.94, TLI = 0.92; *R*^2^ = 0.85).

**Table 2:** Standardized estimates from the dynamic panel model using maximum likelihood for the outcome of weekday sleep and mental health.

Outcome		β-coefficient	95% CI	*p*-value
Weekday sleep	Lag weekday sleep	0.09	−0.14 to 0.32	0.450
	Lag mental health	**0.46** ^***^	0.21–0.71	<0.001
Weekday sleep	Lag weekend sleep	**0.33** ^***^	0.17–0.49	<0.001
	Lag mental health	0.11	−0.14 to 0.37	0.372
Mental health	Lag mental health	0.24	−0.02 to 0.50	0.07
	Lag PA	0.0	−0.0 to 0.0	0.406
	Lag weekday sleep	−0.05	−0.17 to 0.07	0.458
	Lag weekend sleep	**0.11** ^*^	0.01–0.21	0.028

Models adjusted for current university status, sex, race/ethnicity, mother’s highest education level, and experimental group. PA, physical activity; 95% CI, 95% Confidence Interval; bold denotes statistical significance, ^*^p < 0.05, ^***^p < 0.001.

## 4. Discussion

The purpose of this study was to examine the bidirectional associations of PA, sleep, and mental health in a sample of young adults participating in an online wellness intervention. This was a secondary analysis of data obtained from an online wellness intervention during COVID-19. After controlling for individual unit effects and time-invariant covariates, weekday and weekend sleep had a bidirectional longitudinal association with mental health. Although PA associated with mental health within the CLPMs, these significant associations were eliminated using the more rigorous analytical approach (ML-SEM). Additionally, weekend sleep showed the strongest autoregressive associations compared to the other analyzed variables. A discussion of these findings is provided further.

Salient results from this study included a bidirectional association between self-reported sleep and mental health across the online wellness intervention. Sleep is a health behavior known to have positive influences on many mental health outcomes (32–33). A healthy quality and quantity of sleep has been associated with lower anxiety and depression in addition to better cognitive functioning (35–36); these improvements in mental health can further lead to better academic performance and work productivity in young adults. Indeed, within the context of the 24-h movement behavior framework, past observational research has suggested sleep is the health behavior that seems to have the strongest associations with variables of mental and emotional health compared to other behaviors such as PA and screen use (38–39). A previous study that performed polysomnography (PSG) found that children with generalized anxiety disorder reported higher rate of sleep problems compared to healthy controls (6). Another recent study found that depression and anxiety were associated with cortisol, a stress hormone, secretion around sleep onset (7). As shown in these previous studies and the current study’s findings, mental health plays a large role in predicting sleep. However, most prior research examining multiple 24-h movement behaviors have been cross-sectional in design and the associations between health behaviors and outcomes have been examined outside the context of an online wellness intervention (21, 40, 41).

A novel aspect of this study was that mental health predicted future weekday sleep during an online wellness intervention. The Repair and Restoration Theory of Sleep posits that sleeping is needed to revitalize and restore important physiological processes that facilitate proper physical and mental functioning (42). A lack of sleep would theoretically lead to less restoration and poorer mental health, including higher levels of stress, that can subsequently lead to poorer sleep and thus initiate a vicious cycle and a bidirectional association between sleep and mental health (42, 43). This theory is supported by research showing that many functions in the body such as muscle repair, tissue growth, protein synthesis, and release of many of the important hormones for growth occur primarily during sleep (42). Indeed, previous work has shown that using ecological momentary assessment, it was found that sleep and daily mood are reciprocally related but varies greatly by individual levels of mental health (44). This finding supports the Repair and Restoration Theory and a bidirectional association because poor daily mood also affects restorative sleep, especially during the pandemic, as COVID-19 related worry was associated with poor sleep in young adults, possibly by disrupting normal sleep habits (longer sleep latency, more sleep disruptions) (45). Our current study showed that mental health was a positive predictor of weekday sleep and weekend sleep positively predicted mental health, suggesting that the bidirectional associations between sleep and mental health may be determined by day of the week in college students. Mental health predicting future weekday sleep has important implications for college students as maintaining good mental health can lead to better sleep that may further positively affect their work and academic performance. Interventions aiming to improve weekday sleep may want to consider using methods to improve college students’ mental health. Various approaches are available to improve mental health in young adults; however, incorporating stress reduction techniques and mindfulness activities into daily routines has been shown as an efficient and effective way to lower anxiety and improve mood (47–48).

Interestingly, weekend sleep positively predicted future mental health. Weekend sleep was also the behavior that was the most stable throughout the intervention, as indicated by the strongest autoregressive coefficients within both the CLPMs and ML-SEM models. These phenomena may be because college students tend not to have academic classes on the weekends and may have off-days at their places of employment. This may facilitate more regularity in sleep behaviors in young adults on the weekends compared to the weekdays; however, previous work has suggested that socioeconomic status and other demographic variables may moderate this association (49).

Healthy sleep during the weekend may improve mental health through facilitating adequate physical recovery in addition to regulating neurochemicals and neurohormones in the brain that further regulate mood (44, 51–52). A previous study found that brief interventions in addressing health habits using a contract and/or a consultation strategy have the potential to influence positive changes in multiple health behaviors (15). Future research may want to focus on employing wellness interventions as well as investigations into the impact of weekday-to-weekend sleep differences on health outcomes among students, as it is found that the sleep differences can lead to negative mental health outcomes (53). Another recent study that performed a bidirectional relationship between sleep and depression demonstrated self-reported low sleep quality was a predictor of depression or depressive symptoms in college students (54). Similarly, it was found that sleep quantity and quality were critical determinants of health and well-being, and the implementation of intervention programs that target depression, stress management, and healthy sleep patterns are necessary in young adults (55). Better mental health, as discussed previously, may also improve weekday sleep. This potential positive cycle between mental health, weekday sleep, and weekend sleep should be targets for future intervention work as the interrelationships may yield additive benefits on health outcomes.

Another interesting finding from this study was that after individual unit effects and time invariant covariates were accounted for, PA did not show any longitudinal association with mental health. Higher levels of PA have previously been shown to associate with lowered anxiety and depression, and better overall mental health within young adults (57–58). However, much of this prior research has been cross-sectional in design and PA has been mostly examined in isolation without consideration of other health behaviors. When other 24-h movement behaviors were considered, prior research has shown conflicting evidence regarding the statistical significance and strength of PA’s associations with mental health variables (59–62). There are studies suggesting a mediated mechanism linking higher PA to better sleep and mental health (63, 64).

Other explanations for the differences in the observed magnitude and significance of these associations may be the specific mental health variables examined, the types of physical activities participated in, environmental factors that influence PA behavior during the time of assessment, different assessment methods for PA (self-report vs. objective/device-based), and research design (59, 65–68). Future research should examine these associations further using objective assessments of PA to determine if different intensities or patterns of PA behavior in different contexts can be a positive determinant of mental health independent of the quality and quantity of sleep.

Limitations included the use of self-report data collection methods for all the analyzed variables that increased risk of recall and social desirability bias. Data were collected on young adults from one US university during the 2021–2022 school year and during COVID-19, during an online wellness intervention; therefore, caution should be used when generalizing the results to younger or older age groups, students from other universities, and during non-pandemic climates. We also did not collect data on potential stressors or mediators of effect on each of the outcomes. This study was a secondary analysis of data collected during the online wellness intervention; therefore, experimental group comparison was not an aim of the study, however, experimental group was used as a time-invariant covariate within the ML-SEM models to control for the number of coaching sessions. There were no exclusion criteria for participating in this study; therefore, certain participant characteristics or conditions may have contributed to selection bias, have confounded the findings and threatened internal validity, and potentially may make it difficult to reproduce the results within other samples of participants. Finally, data were analyzed from a pilot study and thus the obtained sample size was not necessarily powered to detect weak associations or small effect sizes. Future research should examine the current study’s associations using larger samples sizes to determine if the associations observed between PA and mental health can reach statistical significance.

The interconnectedness of sleep and mental-health play large roles in a young adult’s overall wellbeing. Young adults in the wellness intervention self-reported on mental health, sleep, and other health habits *via* Zoom-based peer interactions with health coaches. Current study’s findings revealed that mental health was a positive predictor of weekday sleep, during which weekend sleep positively predicted mental health. The results of this study support prior research in this area regarding the positive bidirectional associations between sleep and mental health. Caution should be made not to overinterpret the results based on the current study’s use of self-report assessments. Caution also must be made when generalizing the results outside the context of an online wellness intervention implemented during a pandemic climate. Interventions targeting weekday sleep should focus on improving mental health and if targeting mental health, weekend sleep can be targeted. Future research should still be undertaken using larger sample sizes and use objective assessments to enhance the internal validity of the results. Self-reported sleep and mental health had a bidirectional association with each other during an online wellness intervention in young adults delivered during COVID-19.

## Data availability statement

The raw data supporting the conclusions of this article will be made available by the authors, without undue reservation.

## Ethics statement

The studies involving human participants were reviewed and approved by University of Utah Institutional Review Board. The patients/participants provided their written informed consent to participate in this study.

## Author contributions

RB: conceptualization, investigation, methodology, formal analysis, writing—original draft, writing—review and editing. AB: data curation, writing—original draft, writing—review and editing. YB: conceptualization, methodology, data curation, investigation, writing—original draft, writing—review and editing. TB: supervision, project administration, writing—original draft, writing—review and editing. JL: investigation, writing—original draft, writing—review and editing. JK: conceptualization, investigation, methodology, project administration, funding acquisition, writing—original draft, writing—review and editing. All authors contributed to the article and approved the submitted version.

## Funding

This research was supported by the Salt Lake County Health Department, Salt Lake City, UT, United States, with software support from the University of Utah Clinical and Translational Science Institute funded by NCATS/NIH (UL1TR002538).

## Acknowledgments

The authors would like to thank the college students who participated in this study, the peer health coaches who delivered the intervention, and the research assistants that helped collect data.

## Conflict of interest

The authors declare that the research was conducted in the absence of any commercial or financial relationships that could be construed as a potential conflict of interest.

## Publisher’s note

All claims expressed in this article are solely those of the authors and do not necessarily represent those of their affiliated organizations, or those of the publisher, the editors and the reviewers. Any product that may be evaluated in this article, or claim that may be made by its manufacturer, is not guaranteed or endorsed by the publisher.
