# Supplementary material for: Bidirectional associations of physical activity, sleep, and self-reported mental health in young adults participating in an online wellness intervention during the COVID-19 pandemic
Source: Front Public Health. 2023 May 31;11:1168702. doi: 10.3389/fpubh.2023.1168702 (PMC10264583; doi:10.3389/fpubh.2023.1168702)
Supplement: Supplementary file 1 [file Data_Sheet_1.docx]

Supplementary Material

## Supplementary Figure

**Supplementary Figure 1.** Example path diagram of the linear dynamic panel-data model using maximum likelihood and structural equation modeling using weekday sleep as the outcome and mental health as lagged predictors. Adapted from Williams et al.^27^ *Note:* Curved arrows are covariances; straight arrows are direct paths; e stands for error/residual in the regression equation; alpha is a latent variable; coefficients for the effects of mental health on weekday sleep are constrained to be the same at all waves (Ts).

**Supplementary Table 1.** Demographic characteristics of the analytic sample (N=89).

| Variable | Level | N (%) |
| --- | --- | --- |
| University Status | Freshman | 25 (28.1%) |
|  | Sophomore | 24 (27.0%) |
|  | Junior | 18 (20.2%) |
|  | Senior | 22 (24.7%) |
| Sex | Male | 21 (23.6%) |
|  | Female | 65 (73.0%) |
|  | Other | 1 (1.1%) |
|  | No Response | 2 (2.3%) |
| Ethnicity | Not Hispanic/Latino | 72 (80.9%) |
|  | Hispanic/Latino | 17 (19.1%) |
| Race | White | 65 (73.0%) |
|  | Black or African American | 3 (3.4%) |
|  | American Indian or Alaskan Native | 1 (1.1%) |
|  | Asian | 14 (15.7%) |
|  | Hawaiian/Pacific Islander | 2 (2.3%) |
|  | Other | 4 (4.5%) |
| Mother’s Highest Level of Education | Less than High School | 3 (3.4%) |
|  | High School Diploma | 4 (4.5%) |
|  | Some College | 30 (33.7%) |
|  | Associate Degree | 10 (11.2%) |
|  | Bachelor’s Degree | 39 (43.8%) |
|  | Unsure | 3 (3.4%) |
| University Athletics | No | 80 (89.9%) |
|  | Varsity Sports | 4 (4.5%) |
|  | Club Sports | 5 (5.6%) |
| Fraternity or Sorority | No | 82 (92.1%) |
|  | Yes | 7 (7.9%) |
